# p16-mediated G0/G1 cell cycle arrest leads to SASP and fibrosis in Fuchs endothelial corneal dystrophy

**DOI:** 10.1038/s41419-026-08425-6

**Published:** 2026-02-02

**Authors:** Mohit Parekh, Yadav Adhikari, Neha Deshpande, Raymond Wong, Marianne O. Price, Francis W. Price, Ula V. Jurkunas

**Affiliations:** 1 Schepens Eye Research Institute, Massachusetts Eye and Ear, Boston, MA USA; 2 Department of Ophthalmology, Harvard Medical School, Boston, MA USA; 3 PriceVision Group, Indianapolis, IN USA

**Keywords:** Cell death, Mechanisms of disease, Senescence

## Abstract

Fuchs endothelial corneal dystrophy (FECD) is an age-related disorder characterized by excessive extracellular matrix (ECM) deposition and loss of corneal endothelial cells (CEnCs), eventually leading to corneal blindness. Despite known environmental and genetic contributors, the roles of aging and hormonal influences, particularly in the predominantly female population, remain underexplored in FECD. This study investigates the impact of chronic exposure to combined ultra-violet (UV-A) light and the oxidized estrogen metabolite 4-hydroxyestradiol (4-OHE2) on healthy CEnCs, primarily focusing on the cellular senescence pathway implicated in FECD pathogenesis. Our results show that prolonged exposure triggers G0/G1 cell cycle arrest through the p16-pRB pathway, inducing a senescence-mediated pro-secretory phenotype. The senescent cells in G0/G1 phase concurrently upregulated the fibrotic and extracellular matrix (ECM) markers indicating a complex relationship between senescence with fibrosis and ECM deposition. Additionally, multiplex analysis to detect senescence-associated secretory phenotype (SASP) after chronic exposure revealed significant upregulation of pathogenic factors such as IL-8 and IL-17, which were attenuated by SB225002 (anti-CXCR2) and secukinumab (anti-IL-17A). Senolytic cocktail of Dasatinib and Quercetin treatment alleviated fibrosis by selectively eliminating senescent cells and improved the survival of healthy cells. This study introduces a novel in vitro model of FECD, revealing the crucial role of cell cycle modulation, senescence and interleukins in the disease advancement and pathogenesis. The findings suggest that targeting senescence and cytokine-driven inflammation could be a promising therapeutic strategy for mitigating FECD progression.

## Introduction

The human cornea is the outermost structure of the eye comprised of a posterior monolayer of hexagonal endothelial cells that are responsible for maintaining tissue hydration and transparency [1, 2]. Disease or dysfunction of human corneal endothelial cells (CEnCs) lead to corneal blindness as CEnCs cannot self-renew when cell loss occurs in vivo [1]. Fuchs endothelial corneal dystrophy (FECD) is the most common endogenous cause of CEnC loss, which along with the cell death, leads to characteristic extracellular matrix (ECM) deposition in the form of corneal guttae that have been considered critical in the aberrant cell-ECM interactions and loss of tissue function [3–6]. FECD, an age-related, multifactorial disease has been shown to result from a complex interplay of genetic and environmental factors [2]. It affects about 4% of the US population over 40 years of age [2], and accounts for approximately 40% of all the corneal transplant procedures worldwide [7]. However, due to inherent risks of allogeneic transplantation, compounded by the cornea donor shortage worldwide [7], it is important to identify alternatives and, preferably, medical solutions to treat FECD. Therefore, it is critical to understand the underlying mechanisms involved in cellular degenerative processes involved in FECD.

Aging and oxidative stress has been identified as a significant risk factor and contributor to the pathogenesis of FECD [2, 8]. Recently, the induction of oxidative stress by UVA exposure to mouse corneas has been shown to recapitulate the morphological and molecular changes of CEnCs dysfunction seen in FECD [3]. Notably, FECD exhibits a pronounced predominance for females [2], and the studies have shown the involvement of estrogen genotoxicity, mainly in the form of catechol estrogens (4-hydroxyestradiol; 4-OHE_2_) and estrogen-DNA adducts, in causing DNA damage, that leads to CEnC loss [9]. Even though genetic, hormonal, and aging factors influence and contribute towards the disease development, the mechanism of how the confluence of these factors affects the post-mitotic cells, resulting in the decades-long degenerative process, is still unknown.

Cellular senescence is an aging and DNA damage-induced phenomenon that is characterized by cell-cycle arrest to prevent the proliferation of damaged cells in replication-competent tissues [10, 11]. Under normal physiological conditions, CEnCs reside in a quiescent but reversible G0/G1 state [1]. Upon exposure to stress, post-mitotic CEnCs paradoxically re-enter the cell cycle and become arrested in G2/M phase that is dependent on ATM-p53 activation [12, 13]. This leads to initiation of DNA repair pathways that allow cells to resist apoptosis after acute stress [13]. Increased upregulation of p21 (*CDKN1A*), p16 (*CDKN2A*), and NOX4 [6, 14] has been noted in FECD but the mechanisms of how cell cycle re-entry leads to accumulation of senescent cells is unknown. Furthermore, it is unclear what processes govern ECM deposition, i.e. guttae formation, a hallmark of the degenerating CEnCs in FECD. During senescence, cellular overactivation often leads to hypersecretory phenotype, termed senescence-associated secretory phenotype (SASP) that contributes to age-related diseases [15]. Since CEnCs’ death and guttae formation proximally clusters in the central cornea, there is likely a role of SASP in ECM deposition that has not been explored in the past. Furthermore, CEnCs undergo endothelial-mesenchymal transition (EMT) in response to DNA damage, prompting further investigation of the temporal sequence of EMT and senescence in FECD progression [12].

To determine how the cell cycle regulation leads to ECM deposition, we created acute stress (AS) and chronic stress (CS) models of the CEnCs exposed to physiological stressors of UVA and 4-OHE2 and investigated cell cycle-specific onset of EMT, senescence, and fibrosis. Furthermore, we utilized the conditioned media of CS-ed cells to evaluate whether paracrine effects of cellular secretome elicit FECD phenotype in vitro. We detected that after initial stress, G2/M phase of cell cycle leads to upregulation of EMT phenotype along with FECD-specific markers. However, sustained CS causes a progression towards G0/G1 arrest and results in p16-driven activation of senescence and pro-secretory phenotype of inflammatory cytokines and chemokines. Herein, we systematically investigated the role of p16 in the development of fibrosis and identified that together with pro-fibrotic alpha-smooth muscle actin (α-SMA), senescent cells contribute to the development of the unique tissue microenvironment that leads to ECM deposition seen in guttae formation. Given functional heterogeneity of tissue-specific senescence phenotypes, we identified key secretome components associated with chronic cellular degeneration in the eye. Based on the results, we used specific interleukin antagonists in vitro and senolytic compounds in vivo and detected a reduction of both senescence and fibrosis along with ECM components characteristically seen in guttae deposition.

## Results

### Fuchs dystrophy demonstrates a pronounced senescent phenotype and fibrosis

**Fig. 1:**
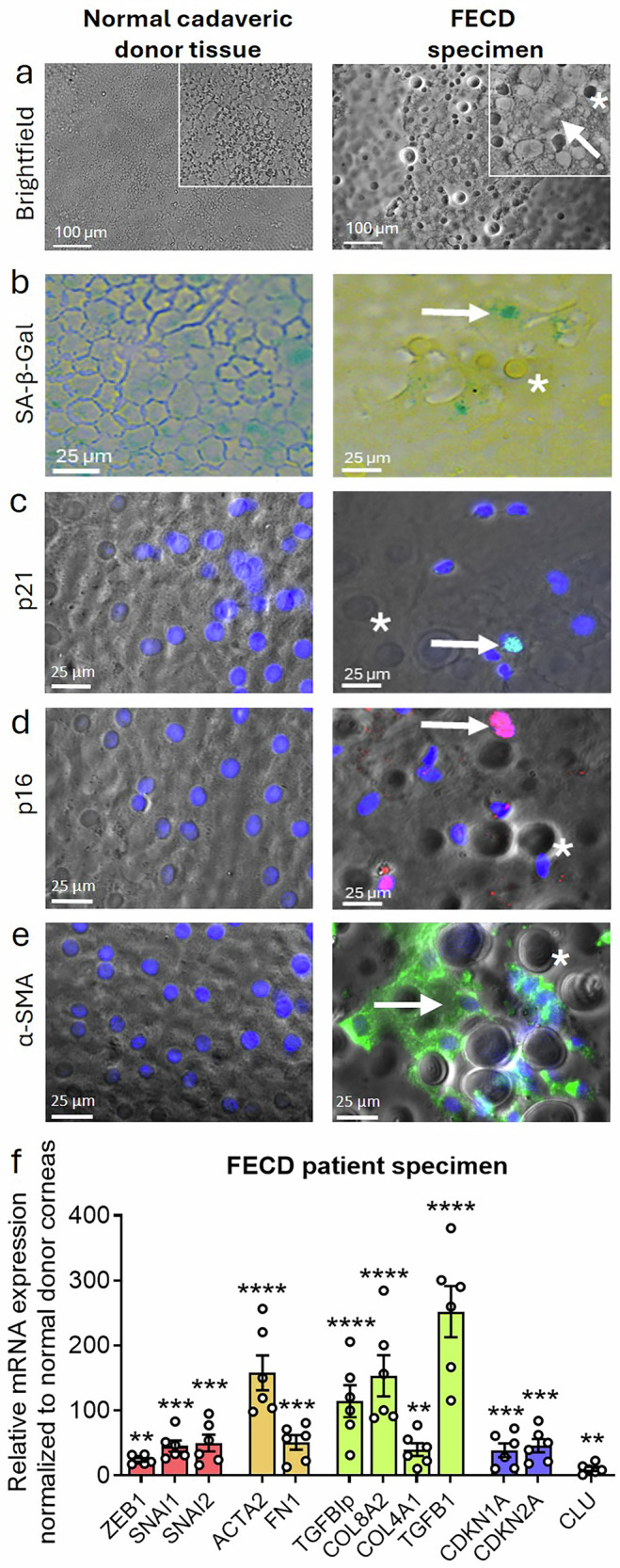
Senescence and fibrosis in Fuchs endothelial corneal dystrophy (FECD) patient specimens. **a** Brightfield imaging of normal human cadaveric donor corneas demonstrating normal hexagonal mosaic of CEnCs, whereas corneal specimens from FECD patient display disrupted intercellular borders with presence of rosette formations (white arrow) and guttae (white asterisk), hallmark morphological features of FECD (figure insets = high magnification). **b** SA-β-Gal staining (green; white arrow) showing presence of senescent cells in FECD specimens compared to the normal tissue. **c** Increased expression of p21 (green; white arrow), and **d** p16 (red; white arrow) positive cells indicative of senescence in FECD tissues compared to healthy controls. **e** α-SMA positive cells (green; white arrow) showing elevated expression in FECD specimens compared to normal controls. Blue: nuclei (Hoechst staining). **f** Quantitative RT-PCR analysis demonstrating upregulation of endothelial-mesenchymal transition (EMT-red bars), fibrosis (orange bars), extracellular matrix (ECM; green bars), senescence (blue bars) and FECD (purple bar) markers in human FECD specimens normalized to normal donor corneal endothelial cells. Data are presented as mean ± SEM; each dot represents one biological replicate. Statistical analysis: One-way Anova with post-hoc Tukey’s multiple comparison test. Statistical significance: **p < 0.01; ***p < 0.001; ****p < 0.0001. Scale bar: **a**) 100 µm, **b**–**e**) 25 µm. Biological replicates: immunostaining (*n* = 3 per marker) and RT-PCR (*n* = 5 per gene). Guttae are highlighted in white asterisk. U untreated, AS Acute stress, CS Chronic stress.

To investigate the potential contribution of lifelong stress exposure to cellular senescence in FECD, we conducted immunostaining and RT-PCR analyses on corneal specimens derived from FECD patients and compared them with healthy donor corneas. Brightfield microscopy revealed a distinct rosette-shaped morphology in FECD specimens, indicative of cellular stress, contrasting with the characteristic hexagonal morphology of normal corneal endothelium (Fig. 1a). Chronic stress in FECD is associated with progressive endothelial cell loss (ECL), leading to compromised intercellular junctions and barrier integrity, ultimately resulting in stromal edema and vision loss. Although ECL is a hallmark of FECD, the contribution of cellular senescence to disease pathogenesis remains underexplored in this aging disorder.

Molecular diagnosis, using senescence-associated beta-galactosidase (SA-β-Gal) staining, detected positive activity of beta-galactosidase (a lysosomal enzyme that becomes active in senescent cells) in FECD specimens, indicative of senescence (Fig. 1b). Immunostaining showed expression of early-senescent marker p21 (encoded by *CDKN1A* gene; Fig. 1c) and mature senescent marker, p16 (encoded by *CDKN2A* gene; Fig. 1d), which are cyclin dependent kinase inhibitors (CDKIs) and regulate cell cycle. Additionally, alpha-smooth muscle actin (α-SMA; encoded by *ACTA2* gene), a fibrosis-associated cytoskeletal protein, was found to be dispersed in FECD specimens (Fig. 1e).

RT-PCR analysis revealed a significant upregulation of *ZEB1* (encoding Zinc Finger E-box Binding Homeobox 1), as well as *SNAI1* and *SNAI2* (encoding the Snail family transcription factors Snail1 and Snail2), which are known repressors of E-cadherin and key inducers of endothelial-to-mesenchymal transition (EMT) in FECD. In addition, elevated expression of pro-fibrotic markers, including *FN1* (encoding Fibronectin-1), which contributes to tissue fibrosis and scarring, was detected in FECD specimens. Components of the TGF-β signalling pathway, including *TGFBIp* (encoding TGF-β-induced protein) and *TGFβ1*, were also upregulated, along with extracellular matrix (ECM) components such as *COL8A2* (encoding the α2 chain of type VIII collagen) and *COL4A1* (encoding the α1 chain of type IV collagen). These findings suggest enhanced ECM deposition associated with guttae formation, a clinical hallmark of FECD. Additionally, *CLU* (encoding clusterin), a molecular marker linked to guttae lesions in the corneal endothelium, exhibited increased expression (Fig. 1f). These findings highlight the contribution of cellular senescence and fibrosis to FECD pathogenesis because of chronic, lifelong exposure to stress, while the multifactorial etiology of the disease facilitates its progression.

### Chronic combined UVA + 4OHE_2_-induced stress drives irreversible senescence via p16-pRB pathway

Environmental, hormonal, and age-related factors are known contributors to FECD pathogenesis; however, the role of combined stressors in disease progression remains poorly understood. To address this, we investigated the effects of individual and combined stressors on early pathogenic events and chronic stress-driven disease mechanisms.

**Fig. 2:**
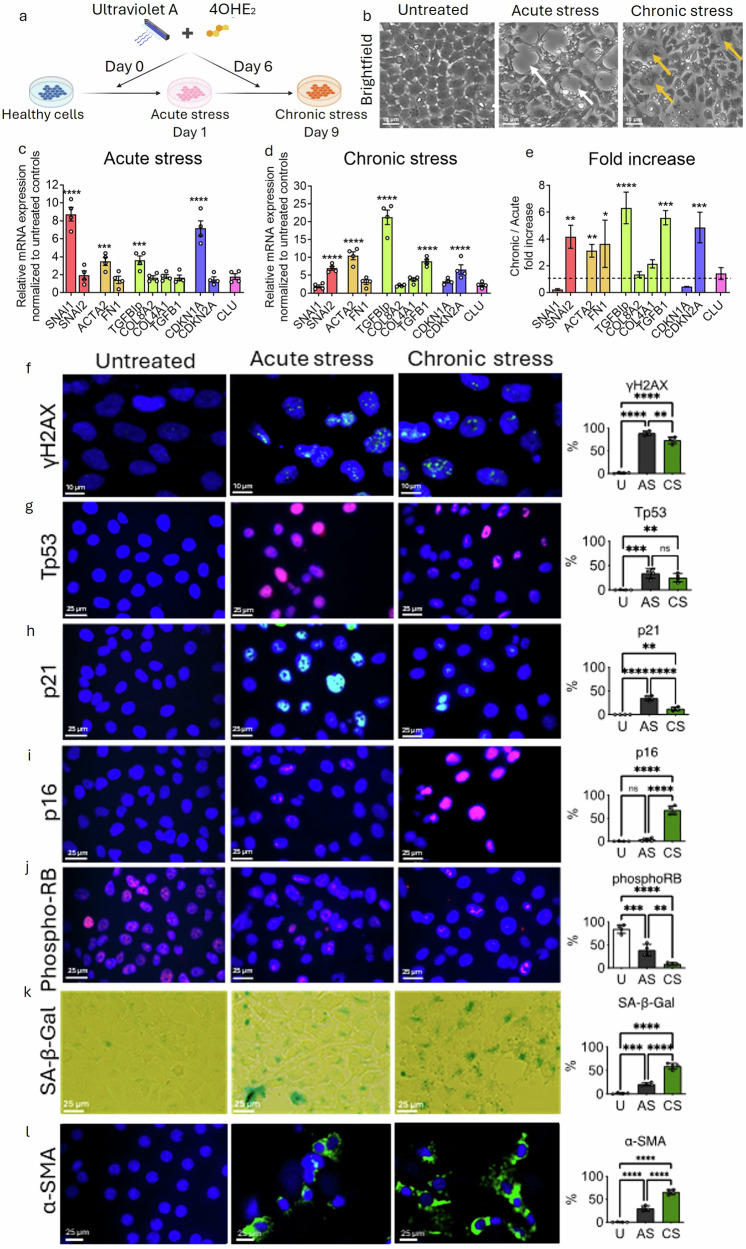
Chronic UVA + 4OHE_2_ exposure induces mature senescence via the p16-pRB pathway and promotes fibrosis in HCEnC-21T cells. **a** Schematic representation of the experimental timeline used to induce acute and chronic stress using concurrent exposure to UVA and 4OHE_2_. **b** Brightfield microscopy images of untreated (U), acute stress (AS), and chronic stress (CS) conditions. AS induced characteristic rosette formations (white arrows), while CS led to senescent-like cell morphology (yellow arrows). Scale bar: 10 µm. **c** Quantitative RT-PCR analysis showing relative mRNA expression levels of EMT (red bars), fibrosis (orange bars), ECM (green bars), senescence (blue bars) and FECD (purple bars) markers in AS and **d** CS, normalized to untreated controls; and **e** fold increase in gene expression in CS relative to AS. Immunostaining for the f DNA damage marker (yH2AX; green) in AS and CS conditions. Scale: 10 µm. Expression of higher (**g**) Tp53 (red), (**h**) p21 (green) in AS, and (**i**) p16 (red) in CS. **j** dephosphorylation of pRB (red) in CS cells. **k** SA-β-Gal staining (green) indicating increased senescent cell population in CS. **l** α-SMA expression (green) observed in AS and CS indicating fibrotic response. Blue: nuclei (Hoechst staining). Scale bar: 25 µm. Data are presented as mean ± SEM; each dot represents one biological replicate. Statistical analysis: **c**–**l** One-way Anova with post-hoc Tukey’s multiple comparison test. Statistical significance: ns=not significant; *p < 0.05; **p < 0.01; ***p < 0.001; ****p < 0.0001. Biological replicates: *n* = 4 for all the analysis.

Initially, the impact of individual stressors on senescence was assessed by subjecting HCEnC-21T cells to a single acute exposure of 4-hydroxyestradiol (4OHE_2_, 20 µM) or UVA irradiation (25 J/cm²). Both treatments independently induced cellular senescence, as indicated by increased p21 expression and SA-β-Gal positivity (Supp. Fig. 1). To evaluate the consequences of concurrent stress exposure, we then treated HCEnC-21T cells with a combination of UVA and 4OHE2 under acute stress (AS) and chronic stress (CS) conditions (Fig. 2a).

Brightfield microscopy revealed distinct morphological changes following stress exposure. AS induced a rosette-like cell morphology (white arrows), while CS resulted in an enlarged, flattened cellular morphology characteristic of a senescent phenotype (yellow arrow; Fig. 2b). Gene expression analysis showed that AS significantly upregulated markers associated with early EMT (*SNAI1*), fibrosis (*ACTA2*), extracellular matrix remodeling (*TGFBIp*), and transient senescence (*CDKN1A*), suggesting early senescence induction (Fig. 2c). However, CS elicited a broader and more robust upregulation of *SNAI2* (a marker of sustained EMT), *ACTA2 (fibrosis marker)*, *TGFBIp*, *TGFβ1 (EMT markers)*, and the mature senescence marker *CDKN2A*, indicative of a more permanent senescent and fibrotic state (Fig. 2d). Notably, *SNAI2*, *TGFβ1*, and *CDKN2A* were exclusively elevated under CS conditions compared to AS, suggesting a shift toward a stable senescence and sustained tissue remodeling (Fig. 2e).

To further explore the link between senescence and FECD pathogenesis, we assessed DNA damage and stress response pathways. Immunostaining revealed increased levels of γH2AX, a marker of DNA double-strand breaks, in both AS (88 ± 4%) and CS (72 ± 7%) groups, compared to untreated controls (2 ± 1%) (Fig. 2f), indicating that both acute and chronic stress induces significant genotoxic stress. Expression of Tp53, a key regulator of the DNA damage response, was elevated in AS (34 ± 9%) and CS (22 ± 9%) relative to controls (1 ± 0.5%), with a higher response observed in AS (Fig. 2g). Furthermore, expression of p21, a cyclin-dependent kinase inhibitor and key downstream effector of Tp53, was elevated in AS-treated cells (36 ± 6%) compared to CS-treated cells (14 ± 4%) and controls (1 ± 1%) (Fig. 2h), indicating Tp53–p21 signalling axis in acute stress.

Interestingly, immunostaining for p16, a canonical marker of irreversible and mature senescence, revealed a significantly higher increase following CS (65 ± 9%) compared to both untreated controls (0 ± 0%) and acute stress (AS; 3 ± 3%) (Fig. 2i). This suggests that sustained stress exposure is required to trigger activation of the p16-mediated senescence pathway. Retinoblastoma protein (RB) phosphorylation is essential for E2F transcription factor release and cell cycle progression. Analysis of phosphorylated RB (pRB) levels demonstrated a reduction of pRB in AS cells and a further decrease in CS cells relative to controls (Fig. 2j), indicating progressive inhibition of cell cycle activity. The observed reduction in pRB, coupled with elevated p16 expression, supports activation of the p16–pRB axis in mediating permanent cell cycle arrest in response to chronic stress. To confirm the senescent state, cells were stained for SA-β-Gal activity. CS-treated cells showed a significantly higher proportion of SA-β-Gal positivity (60 ± 8%) compared to AS (24 ± 4%) and untreated controls (2 ± 1%) (Fig. 2k), further corroborating the onset of a stable senescent phenotype under chronic stress conditions. In addition, α-SMA, a marker of myofibroblast differentiation and fibrosis, was markedly elevated in both AS (30 ± 5.5%) and CS (66 ± 5.4%) groups compared to controls (0.3 ± 0.5%) (Fig. 2l), with significantly higher levels observed in CS. These protein-level findings are consistent with the transcriptional upregulation of EMT- and fibrosis-related genes observed via RT-PCR (Fig. 2e).

Collectively, these results suggest that while acute combined stress triggers transient senescence via the Tp53–p21 pathway, chronic exposure promotes a shift to irreversible, p16–pRB-dependent senescence. This transition is accompanied by enhanced fibrotic remodeling, indicating a critical role for persistent stress in driving pathological features characteristic of FECD.

### Accumulation of p16 after prolonged stress leads to permanent G0/G1 cell cycle arrest and initiates a pro-fibrotic response

**Fig. 3:**
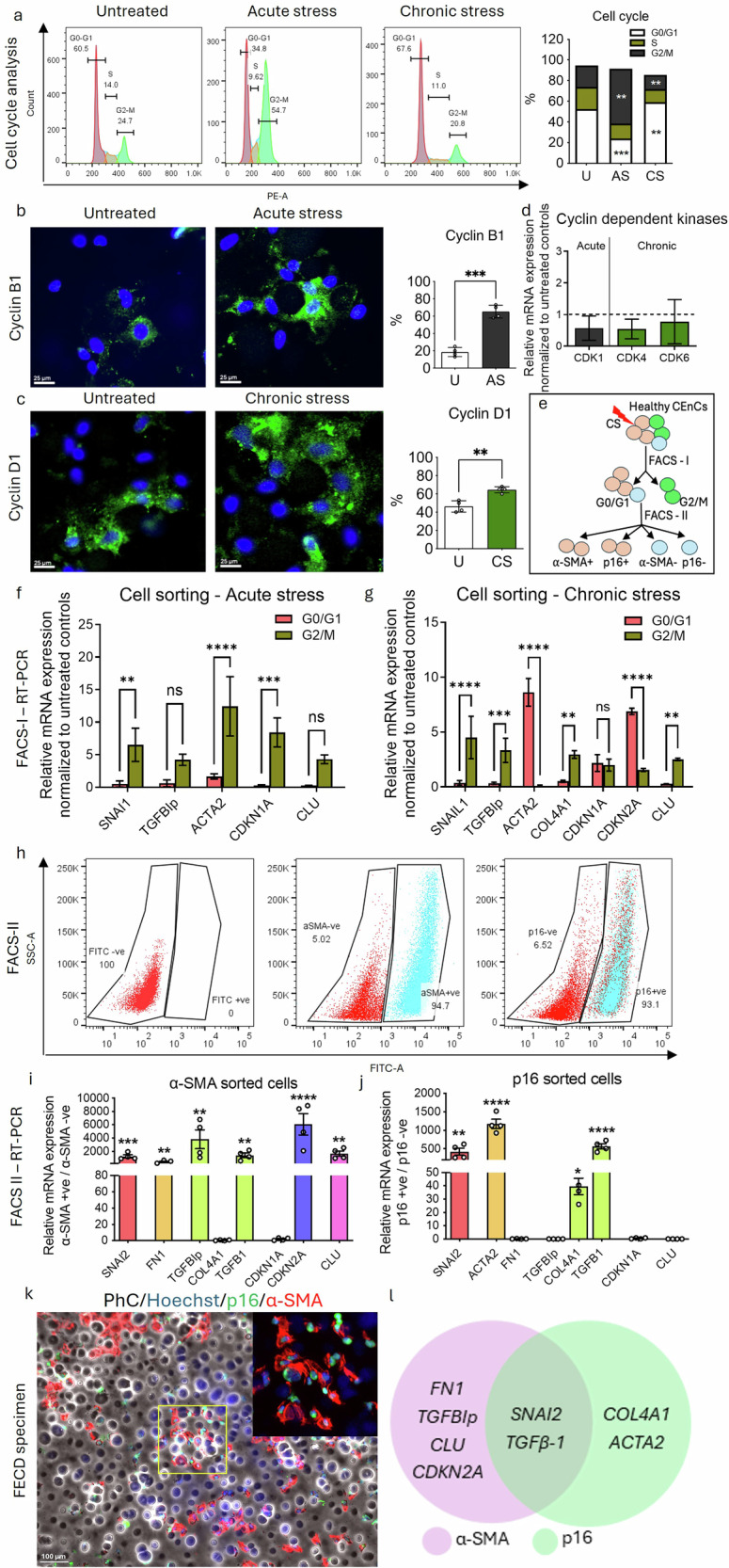
Chronic stress results in irreversible G0/G1 cell cycle arrest mediated by a senescent (p16) – fibrotic (α-SMA) axis. **a** Cell cycle analysis of cells using flow cytometry, treated with UVA + 4OHE_2_ under AS and CS conditions. **b** Expression of Cyclin B1 (green) in AS and **c** Cyclin D1 (green) in CS phase, indicating respective cell cycle phase activity. Blue: nucleus (Hoechst staining). Scale: 25 µm. **d** Cell cycle transition arrest verified by the downregulation of cyclin dependent kinase (CDK)-1 in AS and CDK4/6 in CS conditions normalized to untreated controls. **e** Schematic representation of single (FACS-I) and double sorting (FACS-II) strategies used for downstream analysis. RT-PCR analysis of FACS-I sorted G0/G1 and G2/M cells from (**f**) AS and (**g**) CS conditions normalized to untreated controls. **h** Double sorting (FACS-II) of G0/G1-sorted cells into α-SMA +ve/-ve cells and p16 +ve/-ve cells subpopulations. RT-PCR analysis of double-sorted (**i**) α-SMA +ve and (**j**) p16 +ve populations, each normalized to their respective untreated controls. **k** co-localization of p16 (green) with α-SMA (red) in human FECD patient specimen. Scale bar: 100 µm; inset shows higher magnification image. **l** venn diagram depicting distinct exclusive gene signatures in α-SMA+ve and p16+ve populations. Data are presented as mean ± SEM; each dot represents one biological replicate. Statistical analysis: **a**, **f**, **g** Two-way Anova with post-hoc Sidak’s multiple comparison test. **b**, **c** Unpaired, two-tailed, non-parametric test with Welch’s correction. **d**, **i**, **j** One-way Anova with post-hoc Tukey’s multiple comparison test. Statistical significance: ns=not significant; *p < 0.05; **p < 0.01; ***p < 0.001; ****p < 0.0001. Biological replicates: *n* = 4 for all the groups.

Cellular senescence is characterized by an irreversible cell cycle exit. While we previously demonstrated that acute UVA exposure induces early senescence in CEnCs via p21-dependent G2/M arrest [12], the impact of chronic stress induced by concurrent UVA and 4OHE_2_ exposure on cell cycle regulation remains unclear. To investigate this, we analysed the DNA intensity of cells after temporal induction stress using flow cytometry. AS exposure induced a higher accumulation of cells in the G2/M phase (53 ± 14%) compared to untreated controls (26 ± 2%; p < 0.01). In contrast, CS exposure promoted a shift toward G0/G1 arrest (68 ± 2%) compared to control (60 ± 2%; p < 0.01) with a concomitant reduction in G2/M (20 ± 1%) compared to AS, reflecting progression into a stable, mature senescent state (Fig. 3a).

Cell cycle progression is regulated by various cyclins and CDKs. The G2/M transition is facilitated by the Cyclin B1/CDK1 complex, which promotes chromosome condensation and nuclear envelope breakdown to initiate mitosis. In AS-exposed cells, Cyclin B1 expression was significantly elevated (65 ± 7% vs. 19 ± 5% in untreated controls; p < 0.001) (Fig. 3b), indicating G2/M re-entry. Conversely, CS led to increased Cyclin D1 expression (65 ± 3% vs. 46 ± 2% in controls; *p* < 0.01) (Fig. 3c). Despite this upregulation, nuclear translocation of Cyclin D1 was impaired under CS, leading to reduced RB phosphorylation, thereby inhibiting cell proliferation and arresting cells in the G0/G1 phase. Consequently, CDK4/6 expression was downregulated, impeding G1-S transition and promoting a permanent arrest in the G0/G1 phase (Fig. 3d).

To investigate cell cycle-specific transcriptional changes, we sorted G0/G1 and G2/M phase cells using fluorescent activated cell sorting (FACS) method (Fig. 3e). Post-AS, G2/M cells showed increased expression of *SNAI1* and *CDKN1A* genes, suggesting early EMT and senescence initiation (Fig. 3f). In contrast, CS induced a robust upregulation of *CDKN2A* in G0/G1 phase (2.9-fold; *p* < 0.001), indicative of p16-mediated cell cycle arrest (Fig. 3g). Additionally, while *ACTA2* expression was predominantly observed in G2/M cells following AS, its expression shifted to G0/G1 phase after CS, indicating a transition toward a mature senescence-associated pro-fibrotic phenotype.

To further characterize the senescence-fibrosis phenotype, we performed double-sorting (FACS II) of G0/G1 cells into α-SMA+ve and p16+ve subpopulations (Fig. 3e). FACS II analysis revealed higher proportions of α-SMA+ (94 ± 5%) and p16+ (93 ± 6%) cells within the G0/G1 populations (Fig. 3h), suggesting a strong correlation between mature senescence (p16) and fibrosis (α-SMA). Gene expression analysis using RT-PCR showed upregulation of *FN1*, *TGFBIp*, and *CLU* markers in α-SMA+ cells (Fig. 3i), while p16+ cells exclusively upregulated *COL4A1*, a component of Descemet’s membrane, suggesting that mature senescent cells may contribute to guttae formation via excessive collagen deposition (Fig. 3j).

To assess a direct correlation between p16 and α-SMA in FECD pathogenesis, we then co-stained FECD patient-derived specimens and observed their co-localization, providing in situ evidence for the senescence-fibrosis association (Fig. 3k). Furthermore, both p16+ and α-SMA+ sub-sorted cells exhibited upregulation of *TGFβ1* and *SNAI2*, supporting a mechanism in which senescence-associated, EMT-driven fibrosis during G0/G1 arrest contributes to ECM remodelling driving FECD pathogenesis (Fig. 3l).

### Mature-senescent (p16 + ) cells secrete senescence associated secretory phenotype (SASP) leading to fibrotic activation via paracrine effect

**Fig. 4:**
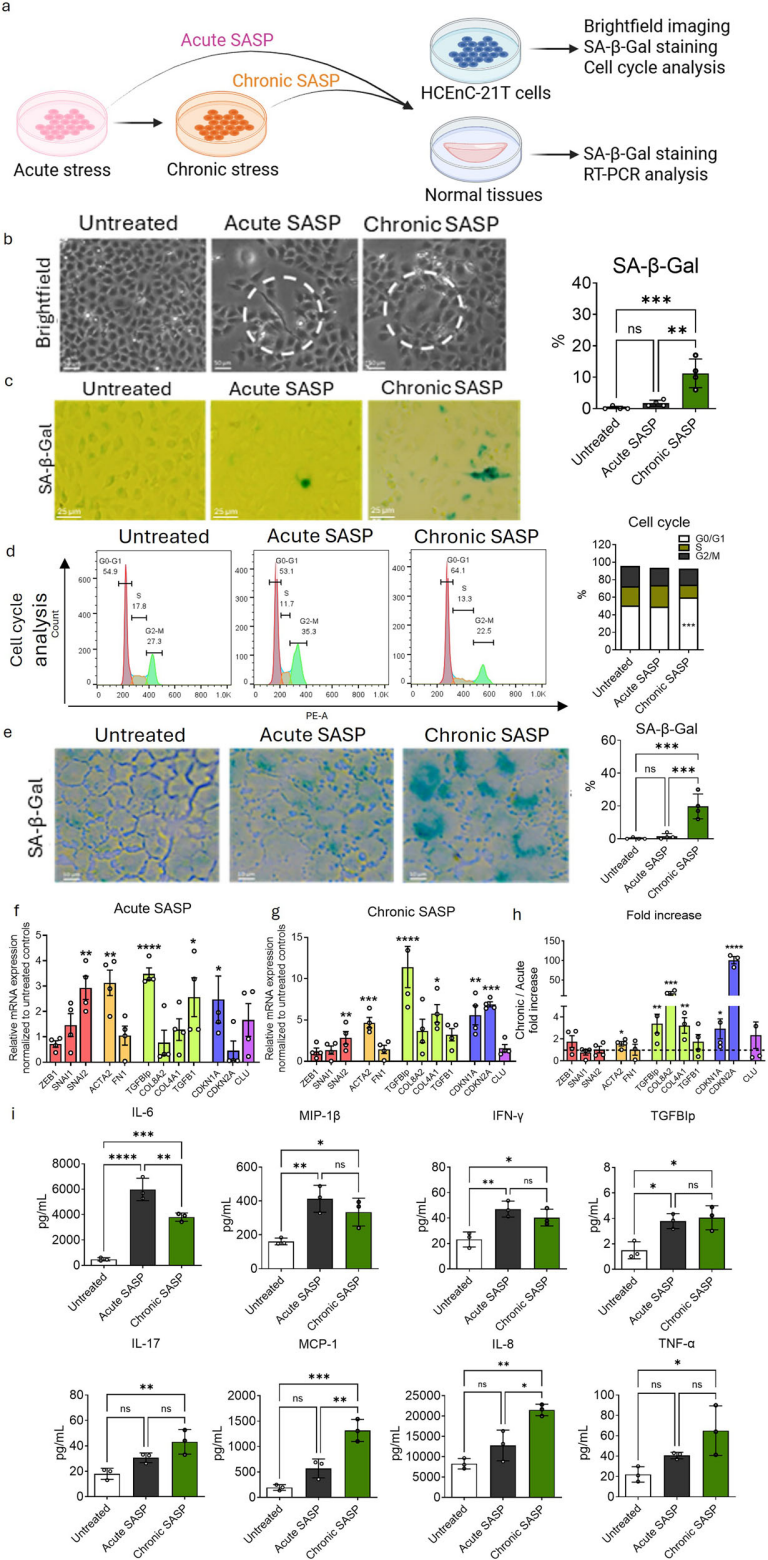
Chronic stressed-induced senescence associated secretory phenotype (SASP) promotes senescence and fibrosis, as observed in FECD. **a** Schematic representation of the experimental pipeline used to evaluate the paracrine effects of SASP derived from acute (Acute SASP) and chronic stress conditions (Chronic SASP). **b** Brightfield images showing the morphology of untreated cells, Acute SASP-exposed cells with EMT-like features (white dotted circle), and chronic SASP-exposed cells with enlarged, flattened senescent-like morphology (white dotted circle). Scale bar: 50 µm. **c** SA-β-Gal staining (green) indicating an increased number of senescent cells exposed to chronic SASP. Scale: 25 µm. **d** Cell cycle analysis showing G0/G1 phase arrest in cells treated with acute and chronic SASP. **e** SA-β-Gal stain showing higher percentage of senescent cells after treating the tissues with acute and chronic SASP, indicative of senescent factors in the chronic SASP. Scale bar: 10 µm. **f** RT-PCR analysis showing relative gene expression of EMT (red), fibrosis (orange), ECM (green), senescence (blue) and FECD (purple) markers after acute and (**g**) chronic SASP, normalized to untreated controls of cadaveric human tissues. **h** Summary plot showing a higher fold increase in fibrotic, ECM and senescent markers observed after chronic SASP conditions. **i** multiplex cytokine array and ELISA results showing increased secretion of SASP factors including IL-6, MIP-1β, IFN-γ, TGFβIp from acute and chronic SASP. Chronic SASP additionally elevated IL-17, IL-8, MCP-1 and TNF-α levels exclusively. Data are presented as mean ± SEM; each dot represents one biological replicate. Statistical analysis: **d** two-way Anova with post-hoc Sidak’s multiple comparison test. **c**, **e**–**i** One-way Anova with post-hoc Tukey’s multiple comparison test. Statistical significance: ns=not significant; *p < 0.05; **p < 0.01; ***p < 0.001; ****p < 0.0001. Biological replicates: *n* = 4 for all the analysis, and *n* = 3 for multiplex and ELISA analysis. Each donor tissue was divided in equal half.

Senescent cells are known to secrete SASP factors that can influence healthy neighbouring cells via paracrine signalling. In the human eye, persistent secretion of SASP by mature senescent CEnCs may accumulate in the anterior chamber and activate transcriptional regulators such as NF-kB, promoting corneal endothelial dysfunction. Although pro-inflammatory cytokines and chemokines such as IL-6 and IL-8 have been detected in the aqueous humor of FECD patients, the precise role of sustained SASP in disease onset and progression remains poorly understood due to lack of robust in vitro models. To address this, we collected conditioned media (SASP) from acutely and chronically stressed CEnCs and evaluated its effects on HCEnC-21T cells and healthy donor corneas (Table 1) to delineate the functional impact of SASP in FECD progression (Fig. 4a).

**Table 1:** Donor characteristics of tissues used for senescence associated secretory phenotype (SASP) study.

	Age (years)	Sex	Death to preservation time (hr:min)	ECD (cells/mm^2^)	COD
**SA-β-Gal + RT-PCR (Control SASP)**	54	M	22:22	1704	Myocardial infarction
	50	M	12:0	3040	Myocardial infarction
	54	F	8:1	2985	Anoxia
	56	M	24:38	2801	Cardiac arrest
**SA-β-Gal + RT-PCR (Acute SASP)**	55	M	23:08	1767	Myocardial infarction
	66	F	8:43	2717	Alcoholic liver disease
	55	M	23:08	1642	Myocardial infarction
	64	F	5:39	3012	Lung Cancer
**SA-β-Gal + RT-PCR (Chronic SASP)**	66	F	8:43	2591	Alcoholic liver disease
	54	F	8:1	3115	Anoxia
	56	F	15:22	1927	Myocardial infarction
	54	F	19:5	2222	Pancreatitis

*M* male, *F* female, *ECD* endothelial cell density, *COD* cause of death, *RT-PCR* reverse transcription polymerase chain reaction, *SA-β-Gal* Senescence-associated beta-galactosidase. Each donor tissue was divided in equal half.

Exposure of HCEnC-21T cells to acute SASP induced an EMT-like- fibroblastic phenotype, whereas chronic SASP exposure resulted in enlarged, senescent-like cells characteristic of mature senescence (Fig. 4b). SA-β-Gal staining demonstrated a significantly higher proportion of senescent cells in cultures exposed to chronic SASP (14 ± 3%) compared to acute SASP (2 ± 1.5%) and controls (1 ± 0.6%; p < 0.01) (Fig. 4c). Additionally, chronic SASP increased the proportion of cells arrested in the G0/G1 phase (60 ± 3%) relative to acute SASP (50 ± 1%) and untreated controls (50 ± 9%), consistent with the onset of cell cycle arrest associated with mature senescence (Fig. 4d).

To validate these findings in human tissue, we exposed normal cadaveric donor corneas to SASP-conditioned media. Chronic SASP exposure significantly increased Sa-β-Gal positive cells (20 ± 7%) compared to acute SASP (2 ± 2%) and control tissues (0.2 ± 0.4%; p < 0.01) (Fig. 4e), supporting the in vitro findings of senescence induced by chronic SASP exposure.

Gene expression profiling of SASP-exposed tissues revealed increased expression of EMT marker (*SNAI2)*, along with *ACTA2, TGFBIp, TGFβ1, and CDKN1A* genes following acute SASP treatment (Fig. 4f). In contrast, chronic SASP induced elevated levels of *SNAI2, ACTA2, TGFBIp, COL4A1, CDKN1A*, and the mature senescence marker (*CDKN2A*) (Fig. 4g). Notably, chronic SASP exposure resulted in a more pronounced upregulation of fibrotic (*ACTA2)*, ECM (*TGFBIp, COL8A2, COL4A1*), and senescence-related *(CDKN1A and CDKN2A*) gene expression compared to acute SASP (Fig. 4h), suggesting a progressive shift from a pro-EMT to a pro-ECM-altering state.

To identify specific SASP components responsible for these effects, we profiled cytokines and chemokines present in the conditioned media using a multiplex assay. Acute SASP was enriched in pro-inflammatory factors including IL-6, MIP-1B and IFN-γ, along with elevated levels of TGFBIp (detected using ELISA), a known ECM remodelling mediator. In addition to these markers, chronic SASP uniquely contained increased levels of pro-fibrotic cytokines (IL-17, MCP-1) and pro-senescent factors (IL-8, and TNF-α) compared to untreated controls (Fig. 4i; Table 2). These findings demonstrate that SASP derived from chronically stressed cells not only reinforce senescence but also promotes fibrotic responses in neighboring healthy cells through a self-sustaining feedback mechanism.

**Table 2:** Senescence associated secretory phenotype (SASP) factors from untreated cells and cells treated with acute and chronic stress with UVA + 4OHE_2_ analysed by multiplex cytokine panel and ELISA.

	Cytokines and chemokines	Untreated (pg/mL)	Acute SASP (pg/mL)	Chronic SASP (pg/mL)	*pValue (Untreated-Acute)*	*pValue (Untreated-Chronic)*	*pValue (Acute-Chronic)*
**Cytokine Panel**	IL-17	15 (1.73)	18.75 (1.25)	21.75 (0.75)	***p*** < ***0.05***	***p*** < ***0.01***	ns
	IL-12 (B-70)	23 (1.73)	22.33 (1.15)	22.66 (1.53)	ns	ns	ns
	IL-7	10 (1.73)	9.66 (0.57)	8.66 (0.58)	ns	ns	ns
	G-CSF	18 (1.73)	16.33 (1.53)	17.33 (0.58)	ns	ns	ns
	IL-10	11.33 (2.31)	12.17 (1.04)	11.0 (1.1)	ns	ns	ns
	MCP-1	229 (20.52)	137.5 (39.31)	1526 (32.91)	***p*** < ***0.05***	***p*** < ***0.001***	***p*** < ***0.001***
	IL-4	10 (1.73)	11.33 (2.52)	10.17 (1.61)	ns	ns	ns
	IL-8	22.33 (7.09)	27.33 (12.74)	74.66 (5.51)	ns	***p*** < ***0.01***	***p*** < ***0.01***
	IL-13	14 (1.73)	13.67 (0.58)	14.0 (1.1)	ns	ns	ns
	IL-6	35.5 (2.5)	51.75 (2.25)	44.38 (0.63)	***p*** < ***0.001***	***p*** < ***0.01***	***p*** < ***0.05***
	IL-1B	10 (1.73)	9 (1)	8.67 (0.58)	ns	ns	ns
	MIP-1B	24.5 (2.5)	29.38 (0.63)	36.38 (0.38)	***p*** < ***0.05***	***p*** < ***0.001***	***p*** < ***0.05***
	IL-5	55.5 (1.5)	60 (14.19)	63.75 (8.75)	ns	ns	ns
	IFN-y	26.5 (1.5)	45.38 (2.48)	43.73 (2.5)	***p*** < ***0.001***	***p*** < ***0.001***	ns
	GM-CSF	28 (2)	35.63 (0.63)	34.67 (3.75)	***p*** < ***0.05***	***p*** < ***0.05***	ns
	TNF-alpha	12.3 (2.52)	15.3 (2.51)	12.3 (1.53)	ns	***p*** < ***0.05***	ns
	IL-2	11.9 (2.6)	14.8 (4.2)	15.4 (1.9)	ns	ns	ns
**ELISA**	TGFBIp	0.11 (0.06)	0.22 (0.01)	0.20 (0.02)	***p*** < ***0.05***	***p*** < ***0.05***	ns

The data are expressed as mean(SD).

Bold=statistically significantly different.

### Pharmacological inhibition of CXCR2 and IL-17A mitigate SASP-driven fibrosis

IL-17A (IL-17 ligand) promotes EMT by inducing TGFβ production [16] and activating fibroblasts, while IL-8, which binds to CXCR1/2 receptors, induces DNA damage–mediated senescence. Given the presence of these cytokines in chronic SASP and their known roles in inflammation and fibrosis, we investigated the therapeutic potential of targeting IL-17 and IL-8 pathways in FECD.

**Fig. 5:**
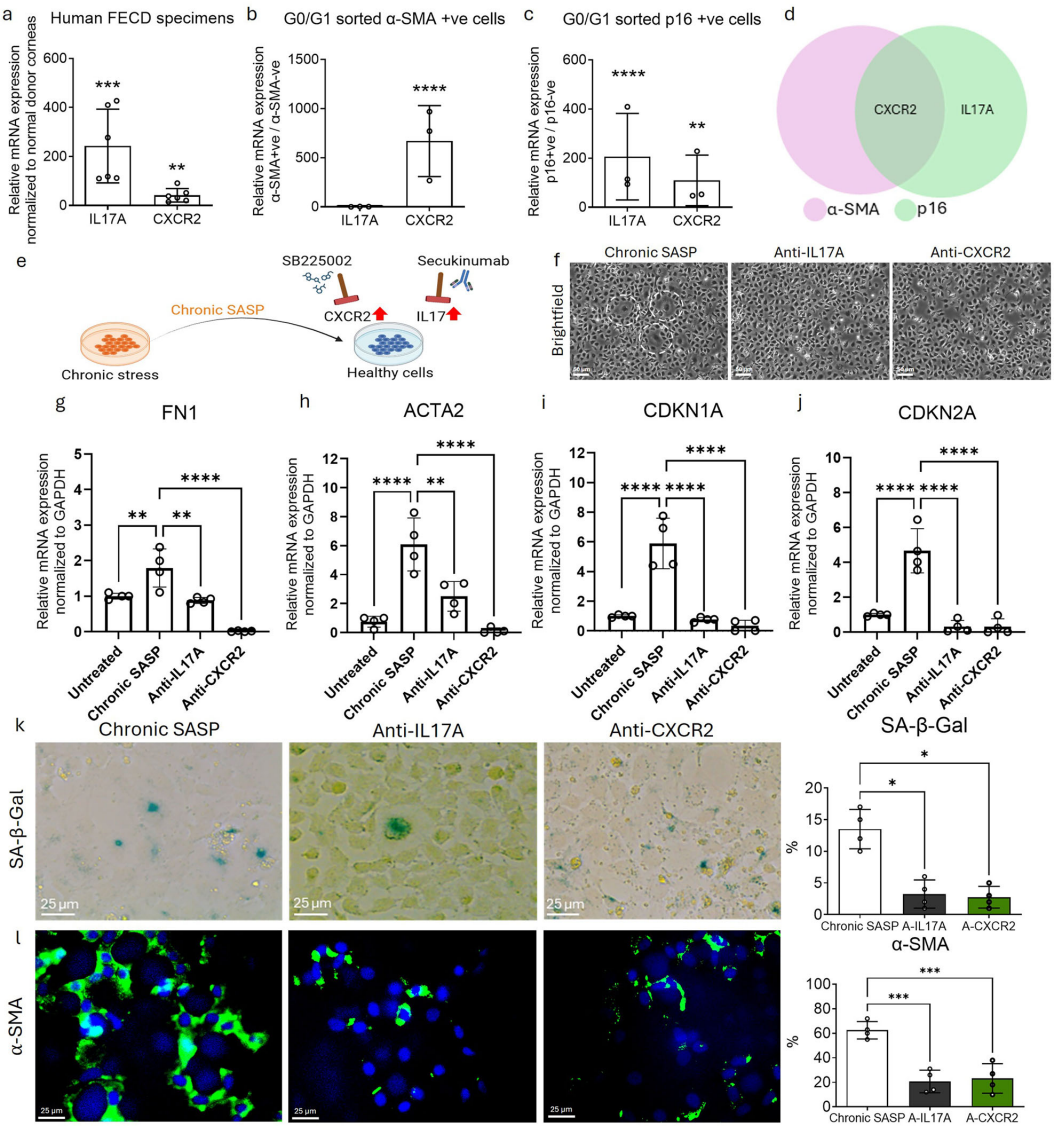
Inhibition of IL-17 and IL-8 attenuates chronic SASP-induced senescence and fibrosis. RT-PCR analysis showing elevated expression of IL17A and CXCR2 in (**a**) human FECD specimens normalized to normal donor tissue, **b** G0/G1 sorted α-SMA+ve, and **c** G0/G1 sorted p16+ve cells, each normalized to their respective untreated controls. **d** Venn diagram showing co-expression of both IL17A and CXCR2 in p16+ve cell populations. **e** schematic of the experimental timeline for IL17A and CXCR2 inhibition by secukinumab (anti-IL17A) and SB225002 (CXCR2 antagonist), respectively. **f** brightfield images showing the morphology of HCEnC-21T cells treated with chronic-SASP (senescent cells marked with white-dotted circle) in the presence or absence of IL17A or CXCR2 inhibitors. Scale bar: 50 µm. RT-PCR analysis showing expression of (**g**) *FN1*, **h** *ACTA2*, **i** *CDKN1A*, and **j** *CDKN2A* genes after treatment with anti-IL17A and anti-CXCR2, compared to untreated controls. **k** SA-β-Gal staining (green) showing reduced senescence in treated cells. (Scale bar: 25 µm). **l** Immunofluorescence showing reduced α-SMA expression (green) following treatment with IL-17A and CXCR2 inhibitors. Scale bar: 25 µm. A-IL17A = Anti-IL17A; A-CXCR2 = Anti-CXR2. Data are presented as mean ± SEM; each dot represents one biological replicate. Statistical analysis: **a**–**c** and **g**–**l** One-way Anova with post-hoc Tukey’s multiple comparison test. Statistical significance: ns=not significant; *p < 0.05; **p < 0.01; ***p < 0.001; ****p < 0.0001. Biological replicates: at least *n* = 3 for all the analysis.

Gene expression analysis of FECD patient-derived specimens revealed a significant upregulation of IL-17A (209-fold, p < 0.001) and CXCR2 (23-fold, p < 0.01), compared to healthy controls (Fig. 5a), indicating activation of these cytokine pathways in FECD pathogenesis. Further, double sorting of α-SMA+ cells from the G0/G1 phase revealed an 870-fold increase in CXCR2 expression (p < 0.0001) (Fig. 5b). Similarly, G0/G1 sorted p16+ cells exhibited elevated expression of CXCR2 (86-fold; p < 0.01) and IL-17A (2092-fold; p < 0.0001) (Fig. 5c), suggesting that these pro-senescent and pro-fibrotic cytokine pathways are strongly enriched in senescent and fibrotic cell populations (Fig. 5d).

To evaluate the functional consequences of cytokine inhibition, HCEnC-21T cells were exposed to chronic SASP in the presence or absence of IL-17A and CXCR2 antagonists (Fig. 5e). Morphological assessment showed improved cellular integrity and reduced senescent morphology following IL-17A and CXCR2 blockade (Fig. 5f). This was accompanied by significant downregulation of key markers associated with fibrosis and senescence such as *FN1* (Fig. 5g), *ACTA2* (Fig. 5h), *CDKN1A* (Fig. 5i), and *CDKN2A* (Fig. 5j). SA-β-Gal staining confirmed a substantial reduction in the proportion of senescent cells after blocking IL-17A (5 ± 3%) and CXCR2 (3 ± 1%) compared to untreated chronic SASP-exposed cells 12 ± 4% (p < 0.05) (Fig. 5k). Moreover, the proportion of α-SMA+ve cells decreased significantly following treatment with IL-17A (21 ± 9%) and CXCR2 (23 ± 12%) antagonists relative to chronic SASP conditions (63 ± 7%) (p < 0.01) (Fig. 5l). These results demonstrate that IL-17 and IL-8 signaling are key drivers of senescence and fibrosis in FECD. Pharmacological blockade of IL-17A and CXCR2 not only restores cell homeostasis but also effectively mitigates the dual pathogenic features of FECD, senescence and fibrosis, highlighting their therapeutic potential.

### Senolytic clearance of p16+ senescent cells attenuates fibrosis and SASP, and restores endothelial morphology in a UVA-induced FECD mouse model

**Fig. 6:**
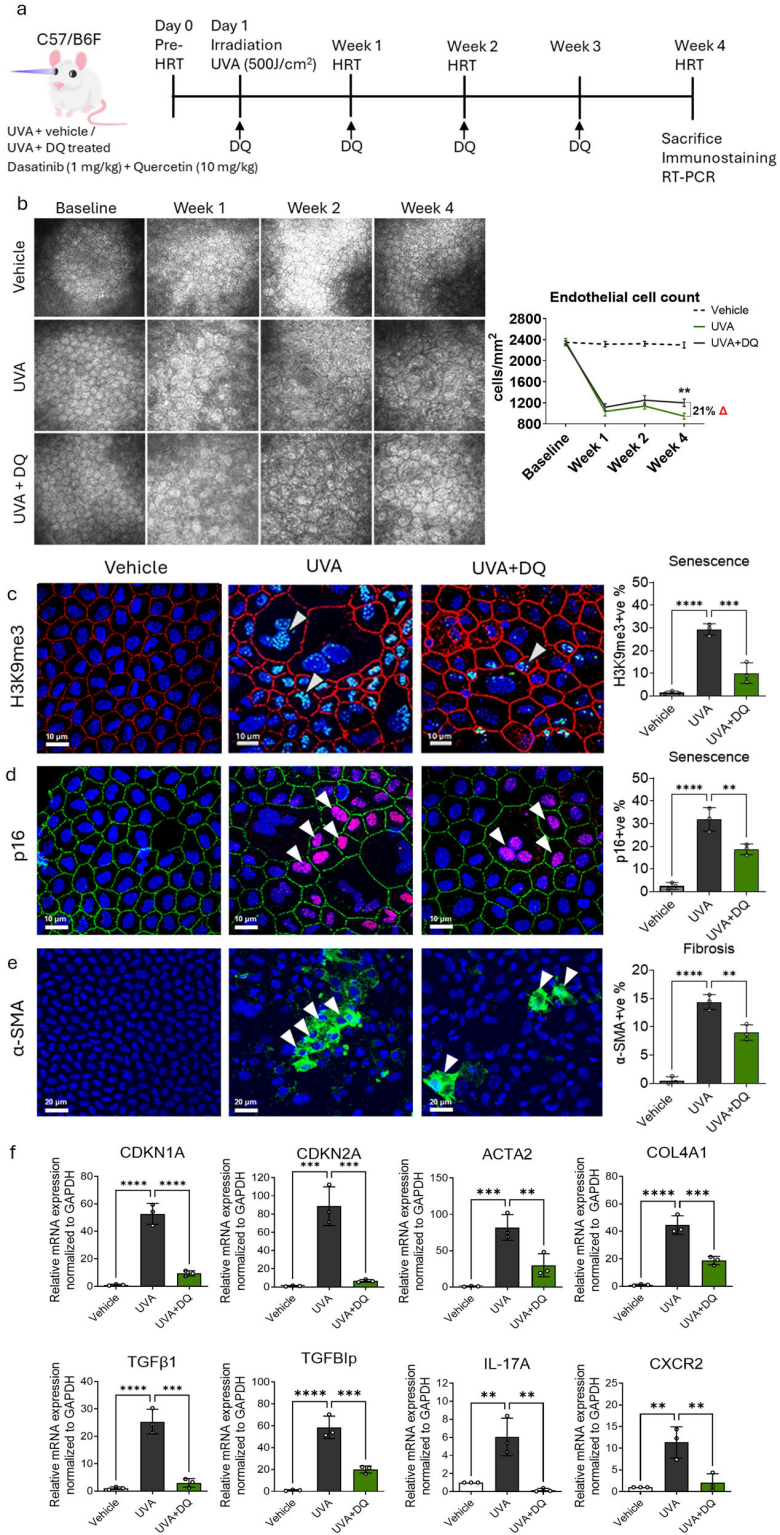
Senolytic treatment with Dasatinib and Quercetin eliminates senescent cells and rescues endothelial cell loss, senescence, and fibrosis in UVA-exposed mice. **a** Schematic of the experimental timeline showing UVA irradiation followed by senolytic treatment with Dasatinib and Quercetin. **b** In vivo corneal imaging by HRT and corresponding corneal endothelial cell density quantification at baseline, and at 1, 2 and 4 weeks post-UVA exposure with and without DQ treatment. **c** Immunofluorescence showing H3K9me3 (senescence marker; green; white arrows) and ZO-1 (intercellular tight junction protein; red) expression in corneal endothelium of UVA-irradiated and DQ-treated mice. Scale bar: 10 µm. **d** immunostaining of p16 (senescence marker; red; white arrows) and ZO-1 (intercellular tight junction protein; green) showing mitigation of UVA-induced senescence (p16) by DQ. Scale bar: 10 µm. **e** α-SMA (fibrosis marker; green; white arrow) showing reduced fibrotic response following DQ treatment. Scale bar: 20 µm. **f** RT-PCR analysis demonstrating the downregulation of senescence, fibrosis, ECM markers and receptors in corneal tissues from DQ-treated mice compared to UVA-only controls. UVA Ultraviolet A light, DQ Dasatinib and Quercetin, HRT Heidelberg Retinal Tomography. Data are presented as mean ± SEM; each dot represents one biological replicate. Statistical significance: **p < 0.01; ***p < 0.001; ****p < 0.0001. Statistical analysis: **b** Two-way Anova with post-hoc Sidak’s multiple comparison test. **c**–**f** One-way Anova with post-hoc Tukey’s multiple comparison test. Biological replicates: at least *n* = 3 for all the analysis.

Several therapies have targeted the selective clearance of senescent cells to alleviate SASP overload in the tissue. Our findings indicate that mature senescence (via p16) is a key driver of fibrosis in chronic stress-induced FECD. To evaluate the therapeutic potential of senescent cell clearance in FECD, we investigated the efficacy of a senolytic cocktail, Dasatinib and Quercetin (D + Q), in a UVA-induced FECD mouse model. Female mice were subjected to corneal UVA exposure 500 J/cm^2^ UVA [3], followed by systemic administration of DQ on day 1 and at weeks 1, 2 and 3. Animals were sacrificed at week 4, and the proportion of senescence and fibrosis markers were assessed by whole-mount immunostaining and RT-PCR (Fig. 6a).

Corneal clarity depends on the transparency which is maintained by CEnC density. In vivo confocal imaging using Heidelberg Retinal Tomography (HRT) demonstrated that UVA disrupted the normal hexagonal CEnC mosaic, accompanied by progressive cell enlargement by week 4 (Fig. 6b). DQ treatment preserved the regular endothelial cell morphology and significantly restored endothelial cell density by 21% relative to UVA-only treated eyes at week 4 (p < 0.01). Confocal microscopy using tight junctional protein (ZO-1) showed extensive multinucleation and nuclear size heterogeneity post-UVA, both of which were reduced with DQ treatment (Fig. 6c), consistent with preserved endothelial structure observed by HRT images.

Next, to determine whether DQ-mediated clearance of senescent cells attenuates fibrosis, we performed whole-mount immunostaining of mice corneas. DQ treatment significantly reduced senescence-associated heterochromatic foci, visualized by the presence of di-methylation of Lys9 on histone H3 (H3K9me3) (10.1 ± 4.5%) compared to UVA irradiation (29.4 ± 2.5%; p < 0.01) (Fig. 6c). The proportion of p16+ve senescent cells was also significantly decreased after DQ treatment (18.7 ± 2.3%) compared to UVA irradiation (31.9 ± 4.5%; p < 0.01) (Fig. 6d). Notably, the number of α-SMA+ve cells (9 ± 1.4%) were significantly reduced by DQ treatment relative to UVA-only controls (14.4 ± 1.3%; p < 0.01) (Fig. 6e), demonstrating the anti-fibrotic effects of senescent cell elimination. Additionally, DQ treatment led to significant downregulation of senescent- (*CDKN1A, CDKN2A, CXCR2*), fibrotic- (*ACTA2*, and *IL17A*) and ECM-associated (*COL4A1*, *TGFBIp, TGFβ1*) markers, indicating a broad suppression of the fibrotic and senescent transcriptomic programs. Pharmacological clearance of p16+ve senescent cells using DQ mitigates UVA-induced endothelial senescence and fibrosis, thereby preserving corneal structure and function and holds a promise for slowing FECD progression.

## Discussion

**Fig. 7:**
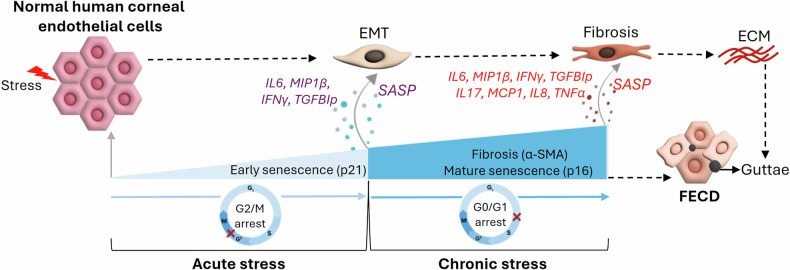
Schematic representation of FECD pathogenesis driven by concurrent UVA and 4OHE_2_ – induced stress. The diagram illustrates the hypothesized progression of FECD, initiated by combined exposure to ultraviolet A (UVA) irradiation and 4-hydroxyestradiol (4OHE2), leading to both acute and chronic stress responses. Acute stress triggers transient cellular responses such as endothelial-to-mesenchymal transition (EMT), while prolonged or chronic stress induces sustained DNA damage, cell cycle arrest, and the accumulation of senescent cells. The resulting senescence-associated secretory phenotype (SASP) promotes fibrosis, extracellular matrix remodelling, and inflammatory cytokine release, collectively contributing to endothelial cell loss, guttae formation, and FECD pathogenesis.

Cellular senescence and fibrosis are common tissue manifestations of aging. Since FECD is a chronic, progressive disorder, there is notion that long-term persistence of senescence, manifested in a cell cycle re-entry and subsequent arrest, promotes development of pro-fibrotic phenotype, as seen in development of other age-related diseases [17–21]. Our previous work demonstrated that UVA light exposure [3] activates DNA damage response in G2/M phase of the cell cycle [13] and triggers endothelial to mesenchymal transition [12] as a response to acute stress. In this study, we show that persistent chronic stress causes cell cycle progression from G2/M to G0/G1 arrest due to activation of CDK inhibitor, p16, which triggers permanent senescence and leads to fibrosis via pro-secretory factor deposition in the extracellular matrix of the post-mitotic cells (Fig. 7). Since SASP is known to be a crucial factor in activating senescence via paracrine signaling, we detected novel secretory factors in the chronic stress model of FECD, including pro-senescent interleukin IL-8 and pro-fibrotic interleukin IL-17. Furthermore, the FECD tissues showed increased levels of IL-17 ligand, IL-17A, and IL-8 receptor, CXCR2 compared to normal ex vivo controls. Sub-sorting of p16-positive population from the G0/G1 phase after the chronic cell cycle arrest, exhibited elevated levels of IL-17A and CXCR2, along with other ECM and EMT components, suggesting a key role of p16 in fostering pro-fibrotic microenvironment. The senolytic cocktail, DQ, diminished accumulation of both p21 and p16 levels and led to a decrease in the pro-inflammatory cytokine receptor levels and ECM components, indicating a potential therapeutic strategy to delay the fibrosis and progression of FECD.

In our model, sub-sorting of specific markers based on cell cycle analysis after acute and chronic stress provides a temporal relationship in a chronic disease progression and supports the proteomic and genomic alterations in diseased human specimens. In the early stage, the activation of p53 and then p21 lead to early and ‘reversible’ senescence that leads to G2/M cellular arrest along with elevation of *SNAI1* and *SNAI2*, heralding the initial cellular response towards the EMT phenotype in FECD [22, 23]. With repeated DNA damage, rise of p16 and cyclin D1 inhibits cyclin dependent kinase 4/6 (CDK4/6) and prevents RB phosphorylation, which is necessary to uncouple RB from E2F; thus, disrupting the G1 to S phase transition and arresting the cells permanently in G0/G1 phase [24]. These findings suggest that while AS triggers cell cycle reentry into G2/M phase, CS drives cells towards permanent cell cycle arrest, underscoring distinct phase-specific cell cycle alterations during the transition from acute to chronic stress. Although the absolute difference in G0/G1 phase between CS and control (68% vs. 60%) may appear modest in the context of an immortalized cell line, the concurrent and statistically significant reduction in G2/M indicates a biologically relevant redistribution of the cell cycle and dynamic progression from acute arrest to chronic senescence.

To further investigate this late stage of disease progression, we focused on the molecular footprint of the chronically stressed cells based on fibrosis (α-SMA positivity) and mature senescence (p16 positivity). The double sorted G0/G1 and p16+ve cells showed upregulation of collagen IV (*COL4A1*), which is an abundant component of DM and together with collagen VIII (*COL8A2*), a collagen specific to DM, has been shown to be aberrantly expressed in FECD ECM [2, 25, 26]. Although both *COL8A2* and *COL4A1* were upregulated after chronic stress and chronic SASP, *COL4A1* was found to be exclusively present in p16+ve cells, suggesting its role in the abnormal basal lamina deposition at onset to mature senescence. The double sorted G0/G1 and α-SMA cells identified robust increase in *CLU*, a secretory molecular chaperone, and TGFBIp, aberrantly upregulated in dystrophic Descemet’s membrane; indicating that profibrotic stimulus likely drives the secretory footprint characteristic of FECD.

Interestingly, both p16 and α-SMA-positive cells arrested in G0/G1 showed robust increase in upregulation of *SNAI2* and *TGFβ1*. TGFβ1 is abundantly expressed in aqueous humor (AqH) [23, 27–29] and significantly upregulated in FECD (causing for increase in TBFBIp in FECD) [5, 30, 31], and has been shown to be master regulator of tissue fibrosis in multiple organ systems [32]. The pro-fibrotic qualities of TGFβ1 have been tightly linked to direct activation of Snail proteins [33] that in turn lead to transcriptional reprogramming of genes such as fibronectin and N-cadherin involved in ECM remodelling [23, 34]. Furthermore, the close interaction between p16 and both TGFβ and Snail proteins underscores their important effect in modulating cell cycle and cell survival. Overexpression of Snail has been shown to decrease CDK4 and phosphorylation of Rb, thus causing a delay, or halting the transition through cell cycle [35]. Moreover, both TGFβ and p16 have been shown to regulate senescence-induced cell cycle arrest in renal disorders [36, 37]. Specifically, TGFβ induced nuclear translocation of p16 in glomerular endothelial cells, a novel finding that shows that not only p21 but also p16 has a direct relationship with TGFβ in chronic kidney injury in vivo. To the best of our knowledge, our study is uniquely proposing a purported role of p16 in TGFβ -mediated ocular degeneration, as shown in co-sorting experiments and corroborated in a chronic SASP model in vitro and UVA mouse model in vivo.

SASP comprises of an intricate network of cytokines, chemokines, and growth factors that can exert pathological effects. In our model, we observed elevated levels of IL-17, MCP-1, IL-8, and TNF-α in the chronic phase, while IL-6, MIP-1β, IFN-γ, and TGFBIp were consistently present in both acute and chronic phases, indicating that the latter may be responsible for disease onset and progression whereas the former sustains pathogenesis. Moreover, similar factors have also been detected in the AqH of the FECD patients [38, 39]. In support of paracrine effects of AqH microenvironment in FECD, application of SASP to HCEnC-21T cells and normal cadaveric donor tissues was able to induce G0/G1 arrest along with upregulation of p16, p21, TGFBIp and collagens seen in FECD specimens. Previous studies have demonstrated that IL-6, IL-8, IFN-γ, and TNF-α activate the NF-kB [40] pathway, establishing a feedback loop that promotes cellular senescence and inflammation in chronic diseases like rheumatoid arthritis and psoriasis [41–49]. Cytokines from chronic SASP, such as IL-17 and MCP-1, promote macrophage recruitment, fibroblast activation, and excessive ECM deposition leading to tissue scarring, features that are characteristic of fibrotic disorders [50–56]. Chronic stress also induced CXCR2 (IL-8 receptor) and IL-17A (IL-17 ligand) [57, 58] upregulation, both of which were elevated in FECD tissues and p16+ senescent cells, indicating their role in disease pathogenesis. IL-8 promotes senescence through paracrine signalling via CXCR2 [41], while IL-17A, acting through the IL-17RA/IL17-RC receptors, drives fibrosis. Both CXCR2 inhibition with SB225002 and IL-17A neutralization downregulated p16 and p21 and fibrotic signalling, as previously observed in other studies [59–61]. Notably, decreasing the activity of IL-17 has shown to mitigate the activity of TGFβ1 and type 1 collagen expression suggesting a synergistic effect of TGFβ1 with Il-17 [62, 63]. While our cytokine panel did not contain TGFβ1, elevated levels of TGFBIp and MCP-1 in SASP that caused upregulation of TFGBIp and TGFB1 in paracrine fashion, suggests that modulation of interleukin receptors have a potential to alleviate the TGFβ-mediated signalling cascade in the aqueous humor [5, 64].

Based on mounting evidence of senescence involvement in the pathomechanisms of FECD, we investigated whether systemic application of a senolytic cocktail had an effect on p16-driven senescence in vivo. A combination of tyrosine kinase inhibitor Dasatinib (D) and the flavonoid Quercetin (Q) has been shown to target anti-apoptotic signalling pathways in senescent cells, making them susceptible to selective elimination [12, 65–67]. Use of DQ cocktail immediately after UVA irradiation reduced senescent cells by lowering p16 positivity by 41% and a fibrotic response by lowering α-SMA positivity by 37%. This led to a 21% rescue of cell loss at a 4-week time-point after UVA irradiation, indicating the robust pro-survival effect by mitigating senescence systemically. Furthermore, elimination of senescent cells was effective in decreasing main SASP components along with key ECM markers known to be involved in the pro-fibrotic guttae formation in FECD. Despite functional heterogeneity of senescent cells in various target organs, p16 has also been detected to play a central role in idiopathic pulmonary fibrosis; whereas senolytic agent XL888 that specifically targeted p16-positive fibroblasts reduced the fibrotic burden in vivo, similar to the findings in FECD mouse model [68]. Moreover, reduction of p16 and p21 positive cells with another senolytic flavonoid, fisetin, led to the reduction of SASP inflammatory markers in the primary sclerosing cholangitis model, highlighting the importance of targeting the secretome with senolytics [69]. Therefore, the primary goal of therapeutic senolytics is the targeted elimination of SASP-producing cellular burden; thereby, promoting the survival of remaining non-senescent cells.

Clinically visible posterior corneal irregularities, characterised by stromal remodelling and associated with delayed visual recovery, have been identified in FECD [70]. Based on our study, the senescent FECD endothelial cells are likely to contribute not only to guttae formation but also lead to pro-fibrosis via paracrine effects that cause posterior stomal ‘ripples,’ impacting vision. Relatedly, in another clinical study, transplantation of donor grafts with healthy endothelial cells has been shown to reduce pro-inflammatory cytokine levels in the aqueous humor in chronic corneal edema patients [71]. Given that aging disorders, such as FECD, are commonly diagnosed in the 5^th^ to 6^th^ decade of life, targeting senescent cells to extend the functional lifespan of healthy cells presents a potential therapeutic strategy in the newly diagnosed patients.

Future studies are needed to explore whether the knockdown of p16 reduces the senescence-mediated cellular dysfunction in FECD. Buj et al. demonstrated that knocking down p16, which transcriptionally regulates SASP factors (reduced expression of IL-6 and IL-8), thereby decreases the SASP activity in oncogene-induced senescent cells [72]. However, it is important to note that while p16 knockdown may prevent cell cycle arrest and senescence, it could potentially result in uncontrolled cell division [73]. Therefore, the distinct outcomes of ‘eliminating’ versus ‘knocking out’ of p16 should be carefully considered in future studies, particularly in the context of FECD pathogenesis. Furthermore, strategies aimed at inhibiting SASP, such as using senomorphics to block pathways like NF-κB, JAK/STAT, or mTOR, hold promises for mitigating cellular senescence-related diseases and offer a complementary approach to alleviate aging-related pathologies [74]. Early intervention in FECD using senolytics to clear senescent cells before significant guttae formation could reduce endothelial loss and slow disease progression. Further, senescence profiling may facilitate patient stratification in FECD, enabling stage-specific application of anti-cytokine therapy to attenuate pathogenic cytokine release.

The study herein contributes to three key points, i) repeated application of UVA and catechol estrogen causes permanent G0/G1 arrest and ECM deposition, the hallmark of degeneration in FECD, ii) pro-inflammatory interleukins in the secretome cause EMT and senescence via paracrine effect, and iii) p16-driven cell cycle regulation leads to ECM and fibrosis in post-mitotic cells, as shown by co-localization and double sorting experiments. The predominance of p16+ve cells ( > 90%) in the G0/G1 phase further implies that p16+ cells play a significant role in the release of SASP factors, particularly those associated with the chronic phase. The identification of distinct factors in the current study that trigger (IL-6), maintain (IL-8) and sustain (IL-17) disease progression indicates temporal and mechanistic complexity of SASP in FECD pathogenesis. Elimination of p16 by senolytics downregulated the pro-inflammatory secretome and reduced fibrosis in the late-onset mouse model of FECD. The innovative treatments with senolytics and cytokine inhibitors may be pivotal for mitigating FECD progression and highlight the need for further research into novel therapeutic strategies.

## Materials and methods

### Ethical statement and donor characteristics

The study followed the tenets of the Declaration of Helsinki and was approved by the institutional review board of the Massachusetts Eye and Ear, Boston, MA, USA (protocol code 2019P003394 and 09/11/2022 of approval). Normal human donor corneas from cadavers were procured from Eversight eye bank (Ann Arbor, MI) and the de-identified FECD specimens were obtained from Price Vision group (Indianapolis, IN, USA) after written consent from the donor’s next-of-kin or the patient for tissues to be used for research. The donor characteristics are listed in Table 3. Association for research in vision and ophthalmology (ARVO) guidelines for the use of animals in ocular research was followed in this study. C57BL/6 J wild-type female mice (7-15 weeks old) were obtained from Charles River (Boston, MA, USA) or were bred in the animal facility of Schepens Eye Research Institute, Mass Eye and Ear (Boston, MA, USA) and were not randomized for this study.

**Table 3:** Donor characteristics of the tissues used in this study.

	Normal (*n* = 26)	FECD (*n* = 18)
**Age (years) mean** **±** **SD**	58 ± 6	73 ± 7
**Sex (M:F)**	10:16	4:14
**Preservation time (hours and min)**	12 h and 25 min	<4 days
**ECD (cells/mm**^**2**^**) Mean** **±** **SD**	2354 ± 524	N/A

### Cell culture, irradiation and SASP collection

#### Cell culture

Telomerase immortalized normal human CEnCs from a 21 year old male donor (CEnC-21T) were cultured using Chen’s media [75, 76] at 37 °C and 5% CO_2_. Upon 80% confluence, the cells were washed with PBS and irradiated with UVA (25 J/cm^2^) on day 0 (Acute stress – AS) and day 6 (Chronic stress – CS). After each irradiation, the cells were incubated overnight in the recovery media (OptiMEM-I supplemented with 1% FBS) containing 4-OHE_2_ (20 µM) for 24 h. Following overnight incubation, the cells were returned to the recovery media and maintained at 37°C in a 5% CO_2_. The media was replenished every 2 days by replacing 50% cultured media with fresh media.

#### Collection of SASP

Following each stress (AS and CS), the cells were washed and incubated in OptiMEM-I (no serum) for 48 h at 37 °C and 5% CO_2_ to collect the conditioned media / SASP. The SASP was centrifuged at 850 g for 10 min at 4 °C to remove any dead cells or debris [77] and filtered through a 0.2 micron filter. The resultant supernatant was either used to study the effect of SASP on healthy cells / tissues or analysing the SASP factors by multiplex analysis.

#### Ex vivo human tissues

Tissues from normal healthy cadaveric donors were rinsed with PBS, and the Descemet’s membrane-endothelial complex was excised using a standard scoring and peeling method [78]. The peeled tissues were subsequently washed and exposed to the collected SASP for 96 h. The tissues were either stained or used for RT-PCR, as outlined in subsequent sections.

### SA-β-Gal staining

The cells and tissues exposed to the stressors or SASP were stained for SA-β-Gal using senescence histochemical staining kit (Sigma-Aldrich, Burlington, MA, USA) according to the manufacturer’s protocol. The images were acquired using EVOS XL core microscope (Invitrogen, Waltham, MA, USA) and percentage of SA-β-Gal positive cells were manually counted and reported as percentage of the total population.

### Cell cycle analysis, single and double-cell sorting

After each stress, cells were collected using trypsin digestion, washed with PBS and fixed in 70% ethanol for 30 min on ice. After washing, the cells were treated with 100 µg/ml RNaseA and stained with 50 µg/ml propidium iodide (PI). Cell cycle data was acquired using BD LSR II flow cytometer (BD Biosciences, Temecula, CA, USA) with at least 10,000 events per sample. Single cells were gated by measuring forward and side scatter and cell doublets were excluded. Quantification was carried out using FlowJo cell cycle analysis (v10.6.2, FlowJo, LLC).

For Fluorescence-Activated Cell Sorting (FACS) based single cell sorting experiments, G0/G1 and G2/M phase cells were sorted using BD FACS Aria III (BD Biosciences, Temecula, CA, USA) and collected in PBS on ice. These cells were immediately centrifuged at 850 g for 10 min at 4 °C and resuspended in Trizol (Invitrogen) for RT-PCR analysis.

For double cell sorting, the cells obtained from CS were fixed in 70% ethanol and treated with 50 µg/ml RNaseA followed by PI staining (50 µg/ml). The G0/G1 population was sorted in PBS and immediately placed on ice. The sorted cells were immunostained (as per the immunostaining protocol given below) using α-SMA, p16 and p21 antibodies and their respective secondary antibodies (Supp. Table 1). The immunostained sorted cells were subjected to a second round of cell sorting with untreated samples as controls. The G0/G1 sorted cells were further sorted into α-SMA+ve and α-SMA-ve, p16+ve and p16-ve, and p21+ve and p21-ve populations separately. The sorted cells were immediately placed on ice, centrifuged and dissolved in Trizol for RT-PCR analysis, as explained in the next paragraph.

### Quantitative RT-PCR

RNA was extracted from cultured CEnCs or tissues using Trizol or Trizol LS (Invitrogen) respectively, purified by washing in absolute propanol and 75% ethanol sequentially, and collected in nuclease free water to measure the RNA quality and quantity using NanoDrop (Thermofisher, Waltham, MA, USA). Complementary DNA (cDNA) was synthesized by iScript (Bio- Rad, Hercules, CA, USA) in a T100 Thermal cycler (Bio-Rad) at 25 °C for 5 min, 46 °C for 20 min, and 95 °C for 1 min. RT-PCR was performed using TaqMan gene expression assays (ThermoFisher) using Eppendorf Realplex2 epgradient S mastercycler (Eppendorf, Enfield, CT, USA). Results obtained were normalized to GAPDH internal control, and the relative gene expression was quantified as 2^^ΔΔ(-CT)^.

### Immunofluorescence staining

CEnC-21T cells, human tissues, and whole mount mice corneas were fixed in 4% paraformaldehyde (PFA) overnight at 4 °C, permeabilized with 0.1% triton x-100 and blocked with 2% bovine serum albumin (BSA) in PBS. Primary antibodies (Supp. Table 1) were diluted in 1% BSA and incubated overnight at 4°C. The cells were incubated with secondary antibodies for 1-hour at RT. Washing was performed with PBS between each step, and the samples were mounted with vecta-shield (Vector Laboratories, Newark, CA, USA) containing DAPI. The images were acquired under Leica DMi8 microscope or Leica SP8 confocal microscope (Leica, Deerfield, IL, USA). Whole mount mice corneas were imaged using confocal microscopy with Z-stacks used to create a maximum projection image (DM6000S with LCS 1.3.1 software, Leica).

### Enzyme-linked immunosorbent assay (ELISA)

The SASP from AS and CS was collected as mentioned above and used to detect TGFBI (BIGH3) using ELISA method (Thermofisher, Waltham, MA, USA) as per the manufacturer’s protocol.

### Multiplex assay for SASP

The SASP factors collected from AS and CS was detected using Bio-Plex Pro Human Cytokines 17-plex assay (Bio-Rad, Hercules, CA, USA) and the plate was read using a MAGPIX® (Luminex corporation, Austin, TX, USA) machine as recommended by the manufacturer.

### Interleukin inhibition

The expression of IL17A and CXCR2 was assessed in the FECD patient specimens and subsequently in sorted α-SMA+ve and p16+ve cells from the G0/G1 phase (as described above) by RT-PCR analysis. HCEnC-21T immortalized cells were exposed to SASP obtained from chronic stress. Concurrently, healthy cells were treated with 10 µM secukinumab (interleukin-17A ligand inhibitor, Selleckchem, Houston, TX, USA), a monoclonal antibody, combined with chronic-SASP to neutralize the effects of IL17A. In a separate experiment, cells were exposed to a combination of 0.2 µM SB225002 (a CXCR2 antagonist, Medchem express, Monmouth Junction, NJ, USA) along with chronic SASP to evaluate the inhibitory effect of CXCR2 antagonist. After 48 h of culture with SASP and the respective IL-antagonists, cells underwent SA-β-Gal staining, α-SMA staining, and RT-PCR analysis.

### Animal study

In the in vivo study involving C57BL/6 J mice, the left eye was designated as the untreated control, while the right eye underwent UVA irradiation using LED light to deliver a dosage of 500 J/cm^2^ on day 1, followed by 4 weeks of recovery period as described earlier [3]. Central corneal thickness (µm) and the endothelial cell density (cells/mm^2^) was measured before irradiation and at 1, 2 and 4 weeks post-UVA exposure [3]. A single dose of combined Dasatinib (1 mg/Kg) and Quercetin (10 mg/Kg) was administered intraperitoneally on days 1, 7, 14 and 21 after UVA exposure [79]. The vehicle used for the treatment consisted of 5% Tween-80 + 5% polyethylene glycol, 90% 1X PBS. Mouse eyeballs were enucleated, rinsed with PBS, and transported on ice for corneal excision on day 28. The corneas were either fixed in 4% PFA for immunostaining or stored in Trizol for subsequent RT-PCR analysis.

### Statistical analysis

Statistical analysis was carried out using Graphpad Prism v10.0.0 (Graphpad Software Inc, CA, USA) using an unpaired, two-tailed, non-parametric test with Welch’s correction (95% CI), one-way analysis of variance (ANOVA) with Tukey’s post-hoc test, and two-way ANOVA with Sidak’s post-hoc test to determine statistical significance deemed at p < 0.05. All the data were analyzed by blinded investigators, which were confirmed by an unblinded investigator. All the data are reported as mean ± SEM.

## Supplementary information

Supp. Figure 1

Supp. Table 1

Checklist

## Data Availability

The datasets generated during and/or analyzed during the current study are available from the corresponding author on reasonable request.
