# Supplementary figures and images for: p16-mediated G0/G1 cell cycle arrest leads to SASP and fibrosis in Fuchs endothelial corneal dystrophy
Source: Cell Death Dis. 2026 Feb 2;17(1):197. doi: 10.1038/s41419-026-08425-6 (PMC12877065; doi:10.1038/s41419-026-08425-6)

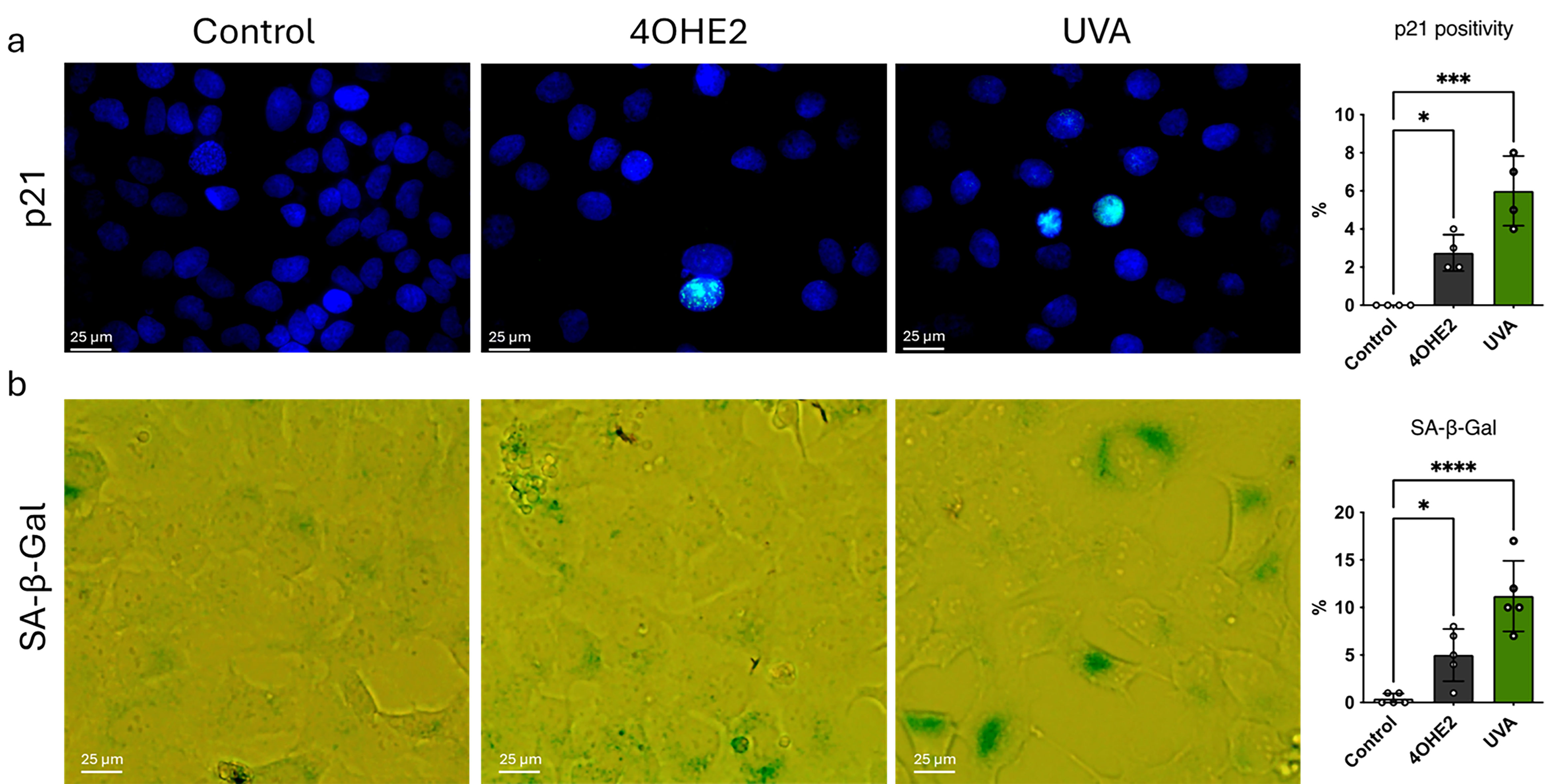

Supplement: Supplementary file 1 — Supp. Figure 1 [file 41419_2026_8425_MOESM1_ESM.png]
