# Supplementary material for: p16-mediated G0/G1 cell cycle arrest leads to SASP and fibrosis in Fuchs endothelial corneal dystrophy
Source: Cell Death Dis. 2026 Feb 2;17(1):197. doi: 10.1038/s41419-026-08425-6 (PMC12877065; doi:10.1038/s41419-026-08425-6)
Supplement: Supplementary file 2 — Supp. Table 1 [file 41419_2026_8425_MOESM2_ESM.docx]

**Supplementary Table**

| **Primary Antibody** | **Dilution** | **Company** | **Cat. No.** |
| --- | --- | --- | --- |
| yH2AX | 1:200 | Sigma-Aldrich | 05-636 |
| p53 | 1:100 | Santa Cruz | SC-6243 |
| p21 | 1:100 | Santa Cruz | SC-5380 |
| p16 | 1:50 | Santa Cruz | SC-1661 |
| pRB | 1:50 | Santa Cruz | SC-271930 |
| Cyclin B1 | 1:200 | Santa Cruz | SC-245 |
| Cyclin D1 | 1:50 | Santa Cruz | SC-8396 |
| H3K9me3 | 1:200 | Abcam | Ab1220 |
| α-SMA | 1:200 | R&D systems | MAB-1420 |
| **Secondary antibody** | **Dilution** | **Company** |  |
| Goat/donkey anti-mouse (Alexafluor 488/594) | 1:500 | Jackson | 115-095-003 |
| Goat/donkey anti-rabbit (Alexafluor 488/594) | 1:500 | Jackson | 711-295-152 |

**Suppl. Table 1.** List of primary and secondary antibodies used for immunostaining.
